# Serial changes of myocardial perfusion imaging in takotsubo and reverse takotsubo cardiomyopathy

**DOI:** 10.1007/s12350-021-02755-y

**Published:** 2021-08-24

**Authors:** Keisuke Miyajima, Kei Tawarahara, Norihito Saito

**Affiliations:** 1 Department of Cardiology, Hamamatu Red Cross Hospital, Hamamatsu, Shizuoka Japan; 2 Department of Cardiology, Saito Clinic, Fujisawa, Kanagawa Japan; 3 Department of Cardiology, Seirei Mikatahara General Hospital, 3453 Mikatahara-cho, Kita-ward, Hamamatsu, Shizuoka Japan

**Keywords:** Takotsubo cardiomyopathy, Reverse takotsubo cardiomyopathy, Myocardial perfusion scintigraphy, Extent score

## Abstract

**Background:**

Takotsubo cardiomyopathy (TTC) shows reversible hypokinesis in the left ventricular (LV) apical-half segment and hyperkinesis in the LV basal-half segment. However, the precise pathophysiological mechanism of TTC is unclear. Therefore, this study sought to clarify the nuclear characteristics, degree of myocardial damage, and serial change of TTC and rTTC using myocardial perfusion imaging.

**Methods:**

We performed myocardial perfusion scintigraphy in 28 patients (TTC: 20, rTTC: 8) using Tc-99m sestamibi and assessed minimum percentage uptake (min-%-uptake), extent score (ES) and summed rest score (SRS) at acute and chronic phases.

**Results:**

Min-%-uptake improved from the acute to the chronic phase (TTC: 54 [48-59]% vs 87 [81-90]%, *P*  < 0.01; rTTC: 60 [55-64]% vs 77 [71-79]%, *P* < 0.01), as did the ES (TTC: 32 [26-41]% vs 0.0 [0.0-6.0]%, *P* < 0.01; rTTC: 16 [12-34]% vs 0.0 [0.0-0.0]%, *P* = 0.02) and SRS (TTC: 4.5 [3.9-5.3] vs 0.0 [0.0-0.2], *P* < 0.01; rTTC: 3.6 [3.3-3.8] vs 0.0 [0.0-0.0], *P* = 0.01).

**Conclusion:**

Tc-99m sestamibi uptake was reduced in hypokinetic regions in the acute phase and improved in the chronic phase. TTC and rTTC may involve a reversible disorder of the myocardial cell membrane, mitochondria, and microcirculation.

**Supplementary Information:**

The online version contains supplementary material available at 10.1007/s12350-021-02755-y.

## Introduction

**Figure 1:**
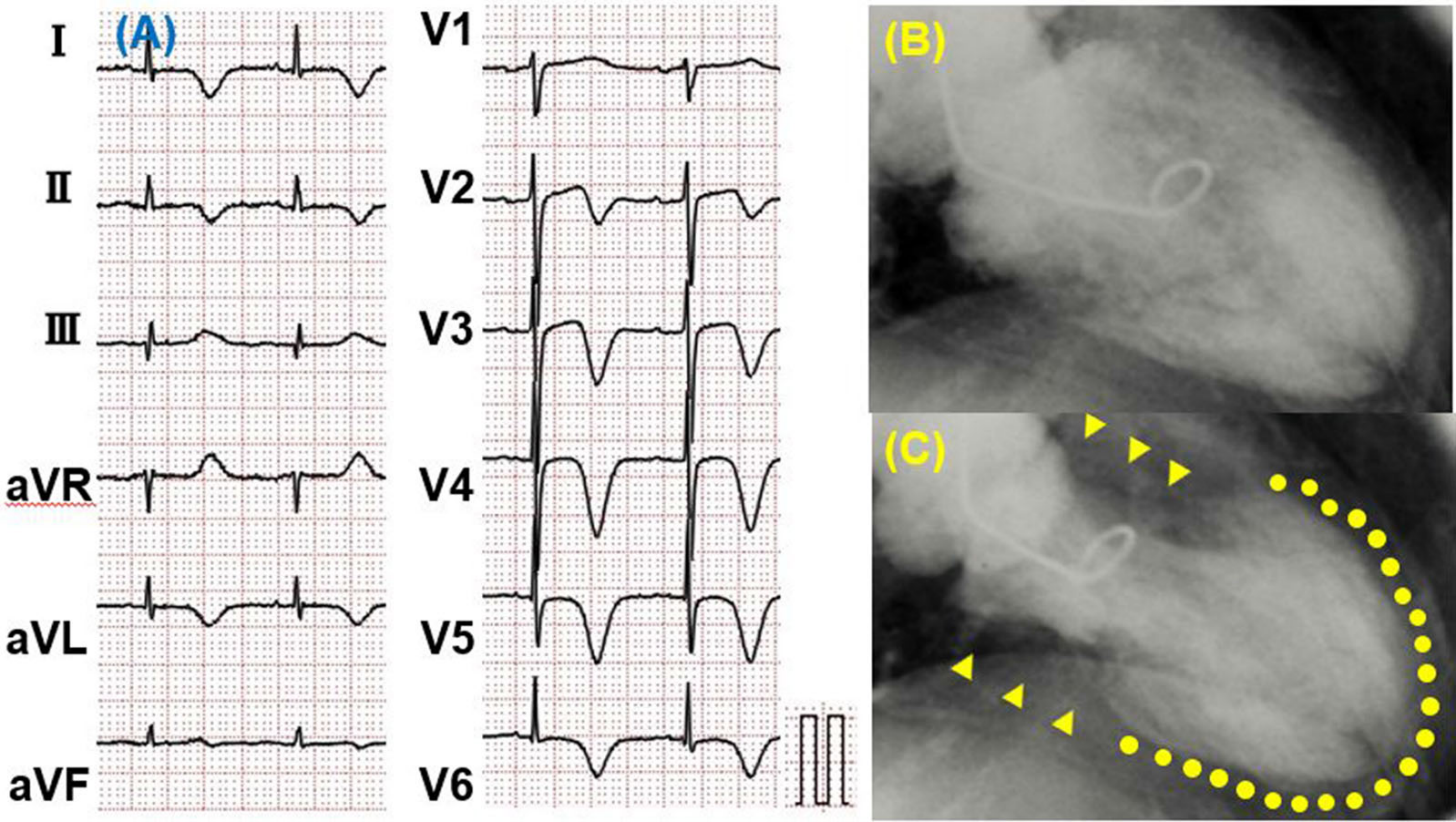
Electrocardiogram (ECG) on presentation to emergency department (**A**) and left ventriculography (LVG) during diastole (**B**) and systole (**C**) in a takotsubo cardiomyopathy (TTC) patient. ECG in a TTC patient showed negative T wave in leads I, II, aVL, V2-V6, and –aVR. LVG in a TTC patient demonstrated severe hypokinesis in the apical segment (**C**, dotted line) and hyperkinesis in the basal segment (**C**, arrow head)

**Figure 2:**
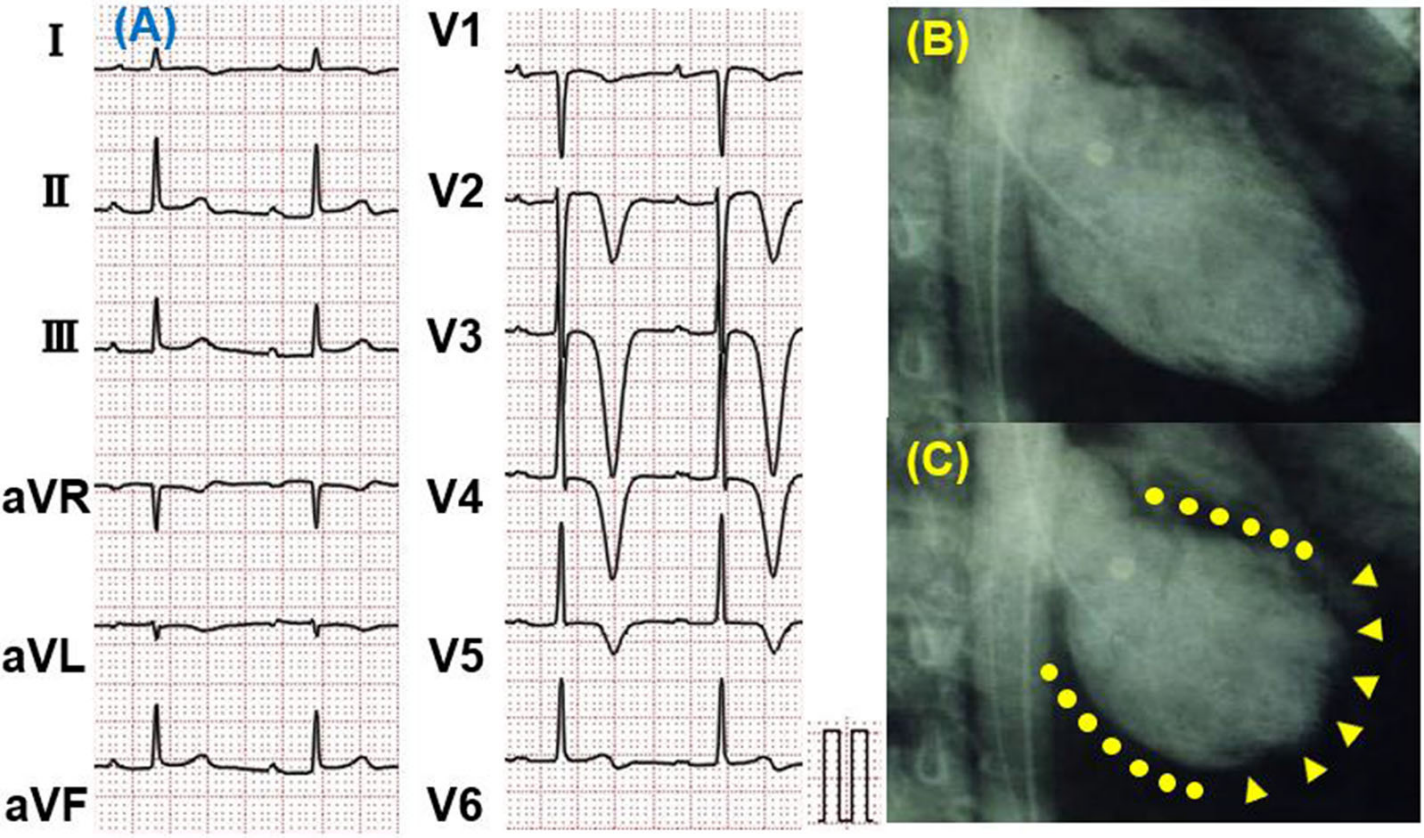
Electrocardiogram (ECG) on presentation to emergency department (**A**) and left ventriculography (LVG) during diastole (**B**) and systole (**C**) in a reverse takotsubo cardiomyopathy (rTTC) patient. ECG in a TTC patient showed negative T wave in leads I, II, aVL, and V1-V6. LVG in a rTTC patient demonstrated severe hypokinesis in the basal segment (**C**, dotted line) and hyperkinesis in the apical segment (**C**, arrow head)

Takotsubo cardiomyopathy (TTC), so-named for its resemblance to a Japanese octopus trap and also known as “broken-heart syndrome,” is accompanied by chest symptoms such as chest pain and shortness of breath following severe emotional or physical stress and changes to an electrocardiogram (ECG) similar to that for acute myocardial infarction (Figure 1A). This disorder, however, has no significant coronary stenosis but demonstrates a “takotsubo shape” with reduced contractions centered in the apex and compensatory basal hypercontraction (Figure 1B, C).^1^-^4^ This left ventricular (LV) wall motion abnormality is transient and normalizes after several weeks. Recently, it has been reported that TTC may be present with atypical morphological variants, including midventricular, basal, and focal types despite similar ECG abnormalities and clinical courses (Figure 2A). The basal type is also referred to as reverse takotsubo cardiomyopathy (rTTC) (Figure 2B, C).^5^

The precise pathophysiological mechanism of TTC is unclear, but there are some hypotheses such as multivessel epicardial spasm,^6^ microcirculatory dysfunction,^7^ vascular endothelial dysfunction,^8^ and catecholamine toxicity of cardiomyocytes.^9^ However, the mechanism of TTC variants, such as rTTC, is completely unknown. Therefore, the purpose of this study was to clarify the nuclear characteristics, degree of myocardial damage, and serial change of TTC and rTTC using myocardial perfusion imaging.

## Methods

### Study Population

**Figure 3:**
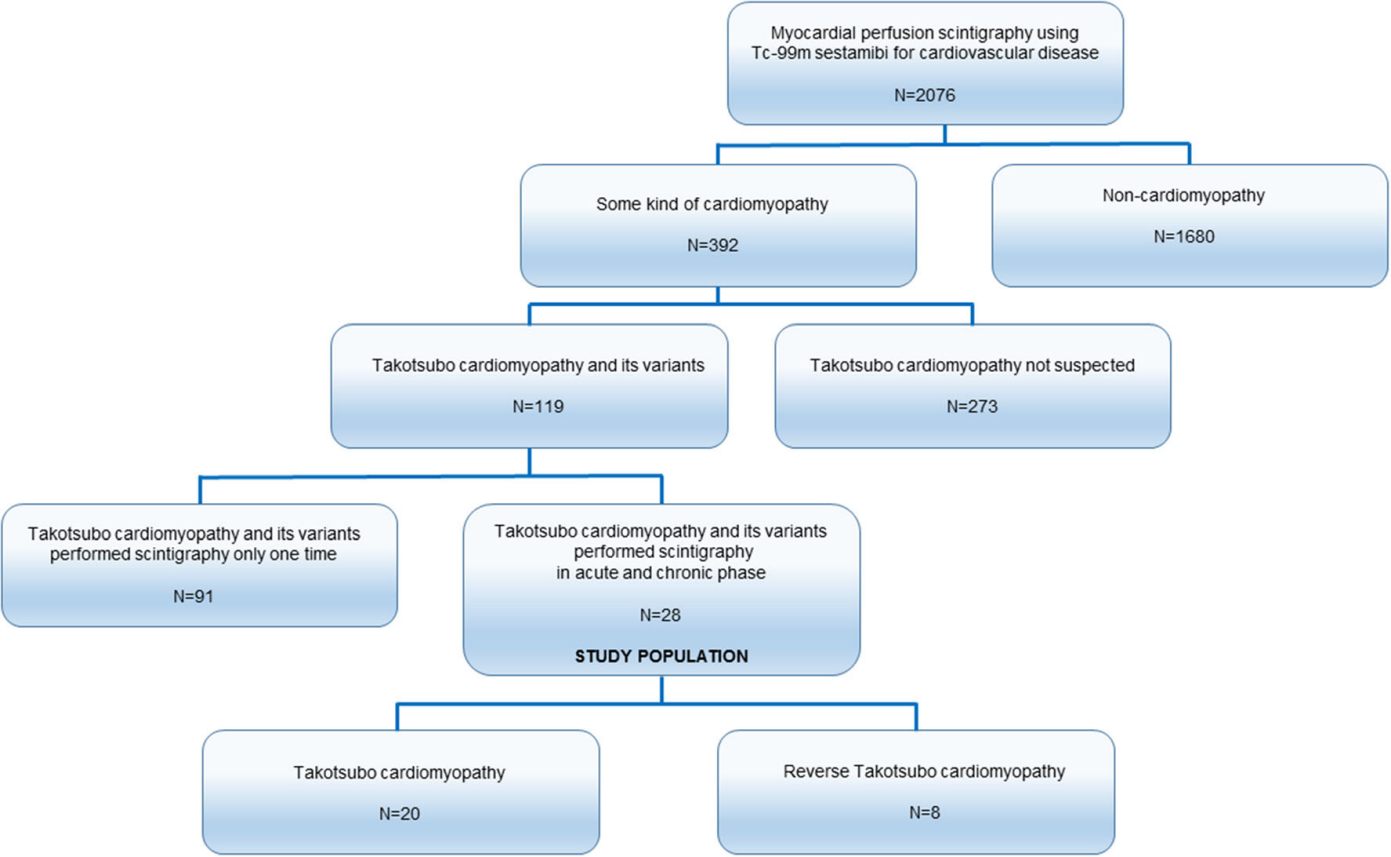
Flow chart depicting inclusion of the study population. We performed myocardial perfusion scintigraphy using Tc-99m sestamibi for 2076 patients between April 2004 and March 2013 in our hospital. 392 patients were diagnosed with a cardiomyopathy, and 30% of them (119 patients) had takotsubo cardiomyopathy or its variant (TTC: 86 patients, rTTC: 10 patients, midventricular: 23 patients). Moreover, 28 patients with takotsubo cardiomyopathy or its variant underwent myocardial perfusion scintigraphy using Tc-99m sestamibi in the acute and chronic phase (TTC: 20 patients, rTTC: 8 patients). *TTC*, takotsubo cardiomyopathy; *rTTC*, reversed takotsubo cardiomyopathy

We performed myocardial perfusion scintigraphy using Tc-99m sestamibi for 2076 patients between April 2004 and March 2013 in our hospital. Thus, approximately 19% (392 patients) were diagnosed with cardiomyopathy, and 30% (119 patients) had takotsubo cardiomyopathy or its variant (TTC: 86 patients, rTTC: 10 patients, midventricular: 23 patients). Moreover, 24% (28 patients) with takotsubo cardiomyopathy or its variant underwent myocardial perfusion scintigraphy using Tc-99m sestamibi in the acute and chronic phase (TTC: 20, rTTC: 8) (Figure 3).

We prospectively enrolled 20 TTC and 8 rTTC patients in our study, on whom we performed myocardial perfusion scintigraphy at the Hamamatsu Red Cross Hospital, Hamamatsu, Japan. Patients were diagnosed with either TTC or rTTC based on the Mayo Clinic criteria: transient hypokinesis, akinesis, or dyskinesis of the left ventricle; regional wall motion abnormalities extending beyond a single epicardial vascular distribution; the presence (not in all cases) of a stressful trigger; absence of obstructive coronary disease or angiographic evidence of acute plaque rupture; new ECG abnormalities (either ST segment elevation and/or T-wave inversion) or modest elevation in cardiac troponin; and the absence of pheochromocytoma or myocarditis.^10^ In addition to these criteria, the cases with mid-apical ventricular hypokinesis and basal ventricular hyperkinesis were diagnosed as TTC. The cases with basal-midventricular hypokinesis and apical ventricular hyperkinesis were diagnosed as rTTC.^11^ In addition, we collected data on patient age, gender, comorbid diseases, clinical presentation, medication, preceding stressful events, and myocardial perfusion scintigraphy findings in both TTC and rTTC groups. Some patients underwent acetylcholine (ACh) provocation test in the chronic phase. Ach was injected using a step-by-step bolus dose of 20/50/100 µg into the left coronary artery LCA and 20/50 µg into the right coronary artery over 20 s. Positive spasm was defined as ≥90% transient stenosis and usual chest symptoms or ischemic changes to the ECG. Scintigraphy findings in the acute and chronic phases in both groups were then compared. Single photon emission computed tomography (SPECT) was performed simultaneously in both groups in the respective acute and chronic phases. In the acute phase, SPECT was performed within a few days after admission (TTC: 3.0 [2.0-3.3] days vs rTTC: 3.0 [2.0-3.0] days, *P* = 0.72), and in the chronic phase, SPECT was performed approximately one month after admission (TTC: 33 [26-37] days vs rTTC: 35 [24-46] days, *P* = 0.63).

This study was conducted in accordance with the guidelines of the Declaration of Helsinki. However, because of the retrospective and observational nature of this study, the need for written informed consent was waived.

### Scintigraphy Protocol

All patients underwent a Tc-99m sestamibi ECG-gated SPECT (rest study only) in the acute phase after hospitalization and in the chronic phase approximately one month later. A dose of 740MBq Tc-99m sestamibi was administered intravenously to each patient, after which each of them received a light meal to improve Tc-99m sestamibi clearance from the hepatobiliary tract. One hour later, SPECT was performed using a digital gamma camera (Shimazu 510R, Shimadzu CO, Kyoto, Japan) equipped with low-energy, high-resolution collimators. Transaxial tomograms of 6 mm-thick sections were processed using the filtered back projection method with the Shepp-Logan filter. The data were reconstructed in the transaxial, short-axial, and vertical long-axial planes. Bull’s eye maps were generated from the short-axial planes. LV function was analyzed using the quantitative ECG-gated SPECT (QGS) software.

### Image Evaluation

**Figure 4:**
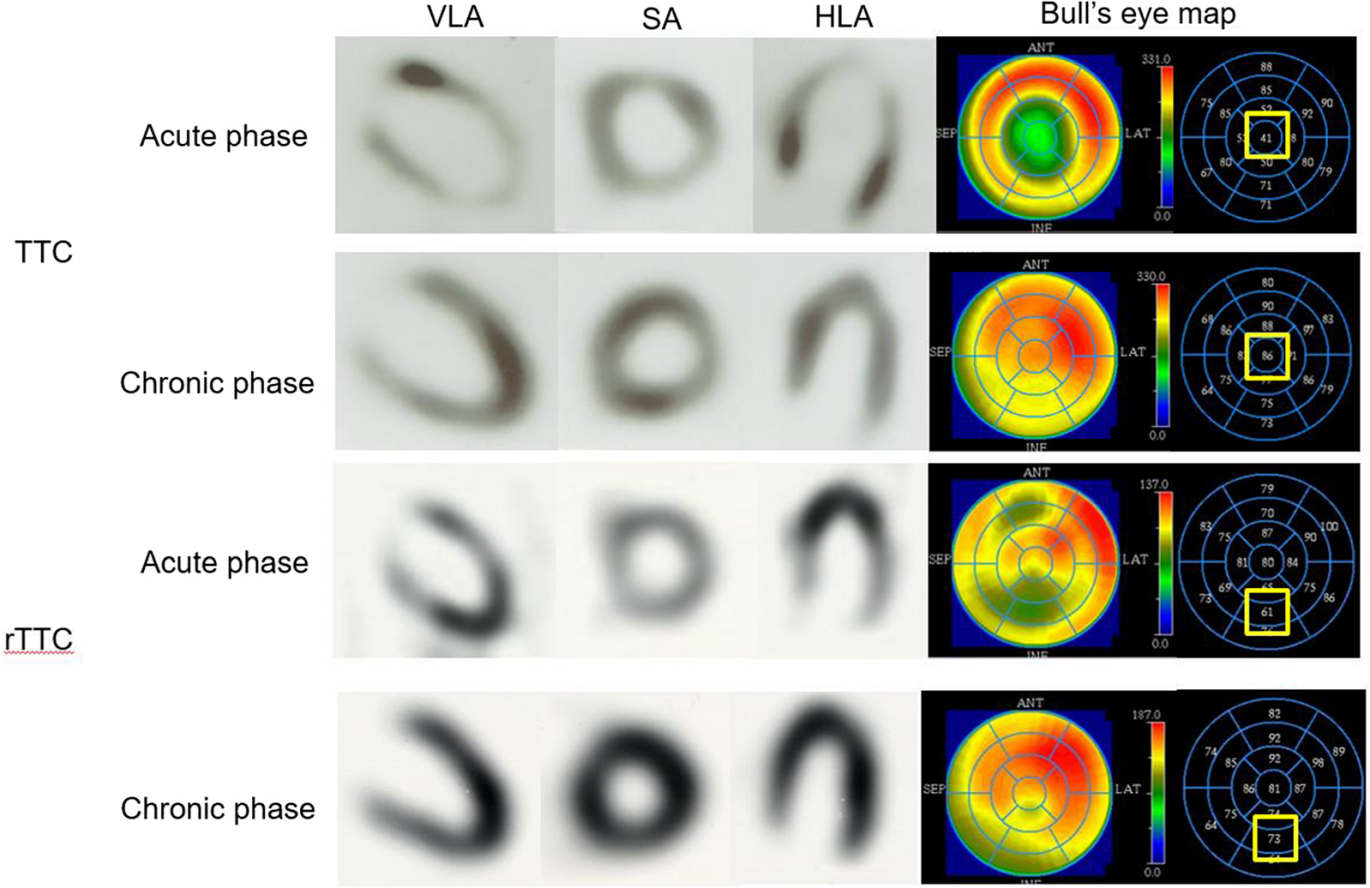
The definition of the minimum-%-uptake (min-%-uptake) in the acute phase and chronic phase. Min-%-uptake was defined the %-uptake in the segment where the accumulation was the lowest in the acute phase on the Bull’s eye map and the same segment in the chronic phase. *TTC*, takotsubo cardiomyopathy; *rTTC*, reversed takotsubo cardiomyopathy; *VLA*, vertical long-axis; *SA*, short-axis; *HLA*, horizontal long-axis; *Ant*, anterior; *SEP*, septal; *LAT*, lateral; *INF*, inferior

**Figure 5:**
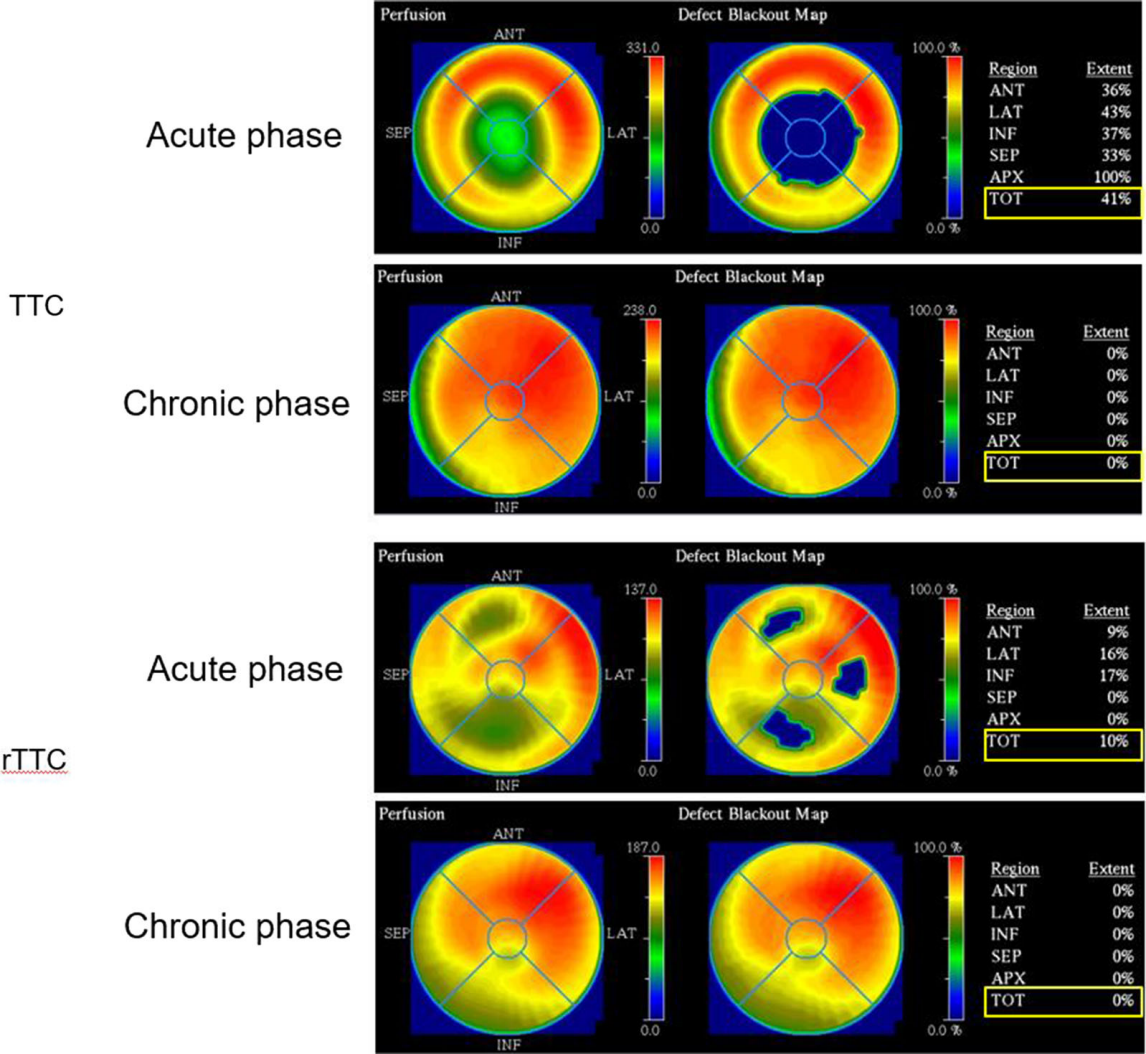
The definition of the extent score (ES). ES was calculated using the Bull’s eye map, with the corresponding lower normal limits at 2.5 standard deviations below the average values derived from the normal data. *TTC*, Takotsubo cardiomyopathy; *rTTC*, reversed takotsubo cardiomyopathy; *Ant*, anterior; *SEP*, septal; *LAT*, lateral; *INF*, inferior; *APX*, apex; *TOT*, total

The LV myocardium was divided into 17 segments on the Bull’s eye map. The Tc-99m sestamibi uptake in each segment was expressed as the percent uptake (%-uptake) in the region with the maximal Tc-99m sestamibi uptake (reference region). We defined the minimum-%-uptake (min-%-uptake) as the segments where the accumulation was the lowest in the acute or chronic phases (Figure 4). The extent score (ES) was calculated using the Bull’s eye map, with the corresponding lower normal limits at 2.5 standard deviations below the average values derived from the accumulated normal database of healthy volunteers (Figure 5). The 17-segment five-point scale was used for visual semi-quantitative assessment of myocardial perfusion, and the summed rest score (SRS) was calculated.^12^

The LV time-volume curve was automatically calculated using QGS software program. The LV end-diastolic volume (LVEDV), LV end-systolic volume (LVESV), and the LV ejection fraction (LVEF) were then derived from the curve. Next, the time-filling rate curve was computed from the first derivative of the time-volume curve (dV/dt). The peak ejection rate (PER) was defined as the minimum dV/dt value divided by EDV (per second). The peak filling rate (PFR) was defined as the maximum dV/dt value divided by EDV (per second). Finally, the one-third mean filling rate (1/3MFR) was calculated as the average of dV/dt values in the first third of the filling time (per second).

### Statistical Analysis

Continuous variables were expressed as median (interquartile range). The Wilcoxon test was used for intragroup comparison between acute and chronic phases, and the Mann-Whitney test was used to compare the TTC and rTTC groups. All categorical variables were expressed as raw numbers and percentages and were analyzed using Fisher’s exact test. A two-tailed *P* value < 0.05 was considered statistically significant. All analyses were performed using R v3.1.1 (The R Foundation for Statistical Computing, Vienna, Austria).

## Results

Baseline characteristics are shown in Table 1. Approximately 55% of TTC and 25% of rTTC patients were asymptomatic. They were diagnosed as TTC or rTTC with abnormal ECG, elevated cardiogenic enzyme, and abnormal LV wall motion after preceding stressful events. We confirmed that all asymptomatic patients were in the acute phase at diagnosis from the combination of findings of ECG, cardiogenic enzyme, and echocardiography. There was no significant difference in patient background between the TTC and rTTC groups. There were more female patients in both groups. Despite in-depth questioning, a quarter of the patients in both groups had no prior psychological or physical stress. Ten patients (50%) in the TTC and two (25%) in the rTTC groups underwent an ACh provocation test. In the ACh provocative test, coronary spasm was provoked in 4 of the 10 (40%) TTC patients and in 1 of the 2 (50%) rTTC patients. Patients provoked with coronary spasms received coronary dilators such as Ca blocker.

**Table 1:** Clinical characteristics

	TTC(N=20)	rTTC(N=8)	*P* value
Age (years)	75 [68-80]	58 [44-83]	0.33
Female gender, N (%)	15 (75%)	6 (75%)	1
Comorbid disease			
Hypertension, N (%)	13 (65%)	3 (38%)	0.23
Diabetes mellitus, N (%)	5 (25%)	1 (12%)	0.64
Dyslipidemia, N (%)	5 (25%)	2 (25%)	1
Chronic kidney disease, N (%)	3 (15%)	3 (38%)	0.31
Smoking, N (%)	4 (20%)	3 (38%)	0.37
Clinical presentation			
Chest pain/dyspnea, N (%)	9 (45%)	6 (75%)	0.22
Abnormal ECG, N (%)	6 (30%)	0 (0%)	1
Elevated cardiogenic enzyme, N (%)	3 (15%)	2 (25%)	0.61
Abnormal left ventricular wall motion, N (%)	2 (10%)	0 (0%)	1
Medication			
Diuretics, N (%)	7 (35%)	2 (25%)	1
Antiplatelet, N (%)	5 (25%)	1 (12%)	0.64
β-blocker, N (%)	2 (10%)	1 (12%)	1
Calcium channel blocker, N (%)	10 (50%)	4 (50%)	1
ACE-I or ARB, N (%)	13 (65%)	3 (38%)	0.23
Statin, N (%)	2 (10%)	1 (12%)	1
Preceding stressful events (%)			
Emotional stressor	4 (20%)	2 (25%)	1
Physical stressor	11 (55%)	4 (50%)	1
No identifiable stressors	5 (25%)	2 (25%)	1

Values are reported as N (%)

*TTC*, takotsubo cardiomyopathy; *rTTC*, reverse takotsubo cardiomyopathy; *ECG*, electrocardiogram; *ACE-I*, angiotensin-converting enzyme inhibitor; *ARB*, angiotensin receptor blocker

The SPECT findings and QGS findings are shown in Tables 2 and 3, respectively. There was no significant difference in min-%-uptake between the two groups in the acute phase (TTC: 54 [48-59] % vs rTTC: 60 [55-64] %, *P* = 0.12). The min-%-uptake in the chronic phase was significantly higher in the TTC than rTTC group (TTC: 87 [81-90] % vs rTTC: 77 [71-79] %, *P* = 0.02). The ES did not differ between the groups during the acute and chronic phases. The SRS in the acute phase was significantly higher in the TTC than rTTC group (TTC: 4.5 [3.9-5.3] vs rTTC: 3.6 [3.3-3.8], *P* = 0.03). The LVEDV, LVESV, LVEF, PER, PFR, and 1/3 MFR did not differ between the groups during the acute and chronic phases (Table 3). There were 3 cases (15%) of TTC and 1 case of rTTC (12%) that had severely decreased LV systolic function with LVEF ≤40% in the acute phase.

**Table 2:** SPECT findings

	TTC (N=20)	rTTC (N=8)	*P* value
SPECT performing day from hospitalization			
Acute	3.0 [2.0-3.3]	3.0 [2.0-3.0]	0.72
Chronic	33 [25-36]	35 [23-45]	0.63
Min %uptake			
Acute	54 [48-59]	60 [55-64]	0.12
Chronic	87 [81-90]	77 [71-79]	0.02
Extent score			
Acute	32 [25-40]	16 [11-33]	0.11
Chronic	0.0 [0.0-6.0]	0.0 [0.0-0.0]	0.37
Summed rest score			
Acute	4.5 [3.9-5.3]	3.6 [3.3-3.8]	0.03
Chronic	0.0 [0.0-0.2]	0.0 [0.0-0.0]	0.08

*TTC*, takotsubo cardiomyopathy; *rTTC*, reverse takotsubo cardiomyopathy; *SPECT*, single photon emission computed tomography

**Table 3:** QGS findings

	TTC (N = 20)	rTTC (N = 8)	*P* value
LVEDV (ml)			
Acute	63 [47-77]	84 [81-92]	0.07
Chronic	46 [42-56]	63 [49-64]	0.19
LVESV (ml)			
Acute	27 [20-37]	41 [31-41]	0.29
Chronic	11 [8.5-16]	21 [16-21]	0.09
LVEF (%)			
Acute	54 [50-60]	59 [54-61]	0.63
Chronic	76 [70-83]	65 [63-72]	0.12
PER (/s)			
Acute	3.4 [3.0-3.9]	2.9 [2.5-3.5]	0.17
Chronic	5.0 [3.3-5.0]	3.2 [3.1-3.5]	0.27
PFR (/s)			
Acute	2.5 [2.0-3.3]	2.2 [2.1-2.5]	0.49
Chronic	2.8 [2.7-3.2]	2.4 [2.1-2.4]	0.06
1/3 MFR (/s)			
Acute	0.92 [0.71-1.2]	1.0 [0.92-1.2]	0.36
Chronic	1.04 [0.84-1.4]	1.2 [1.1-1.6]	0.69

*QGS*, quantitative ECG-gated SPECT; *LV*, left ventricular; *EDV*, end-diastolic volume; *ESV*, end-systolic volume; *EF*, ejection fraction

**Figure 6:**
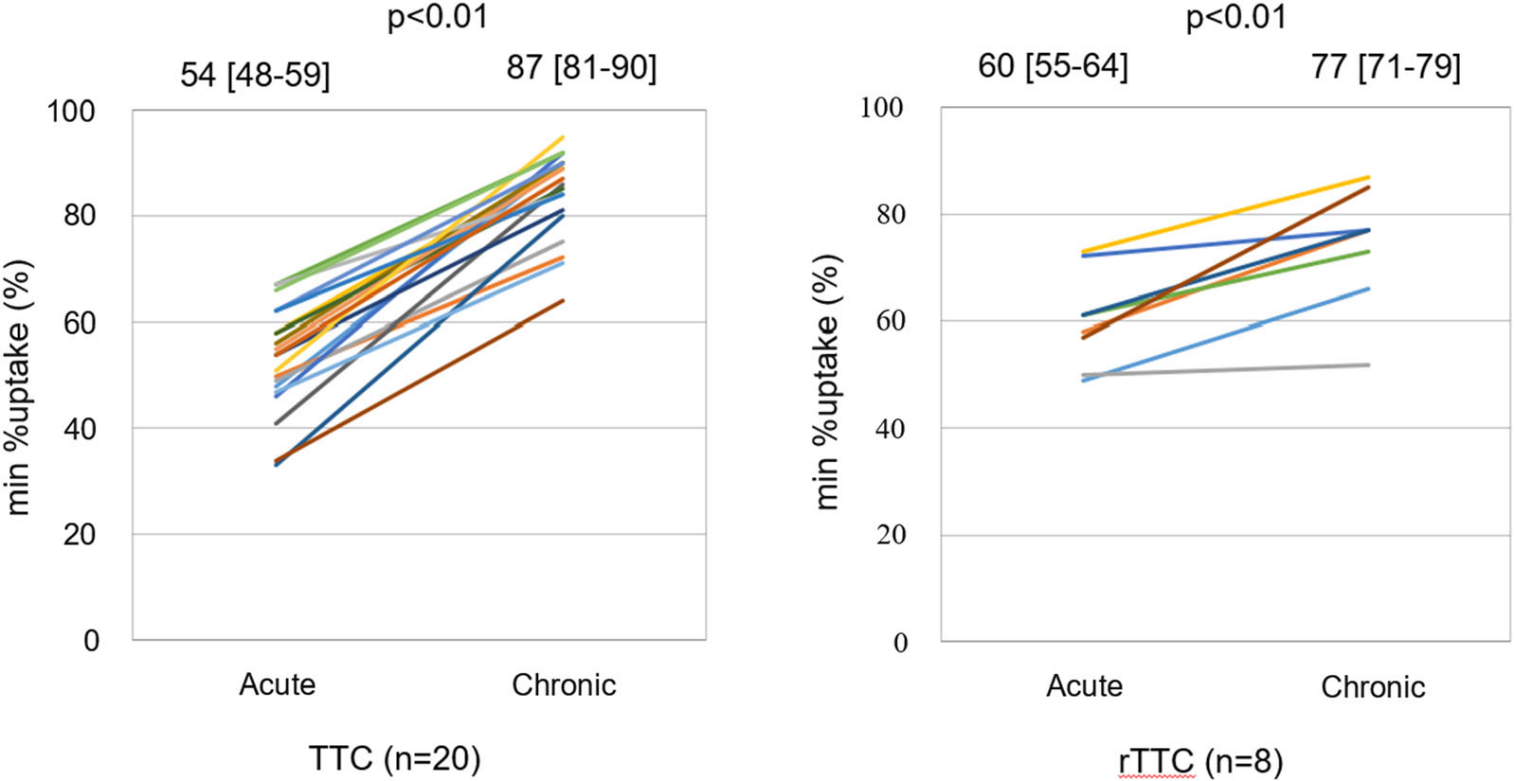
Serial changes of the minimum-%-uptake (min-%-uptake). The min-%-uptake was improved from the acute to the chronic phase in TTC and rTTC group. *TTC*, takotsubo cardiomyopathy; *rTTC*, reverse takotsubo cardiomyopathy

**Figure 7:**
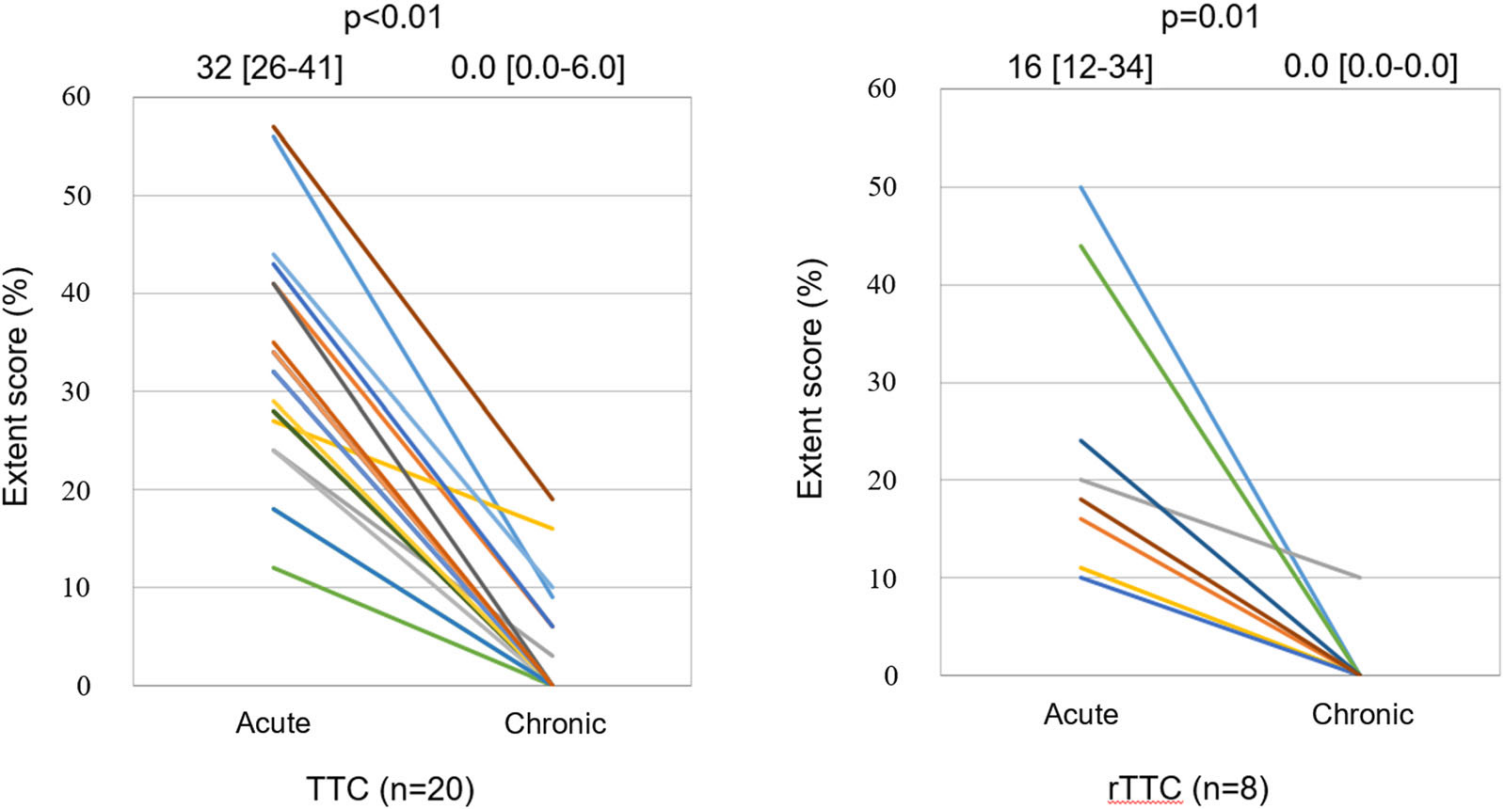
Serial changes of Extent score. Extent score was also improved from acute to chronic phase in TTC and rTTC group. *TTC*, takotsubo cardiomyopathy; *rTTC*, reverse takotsubo cardiomyopathy

**Figure 8:**
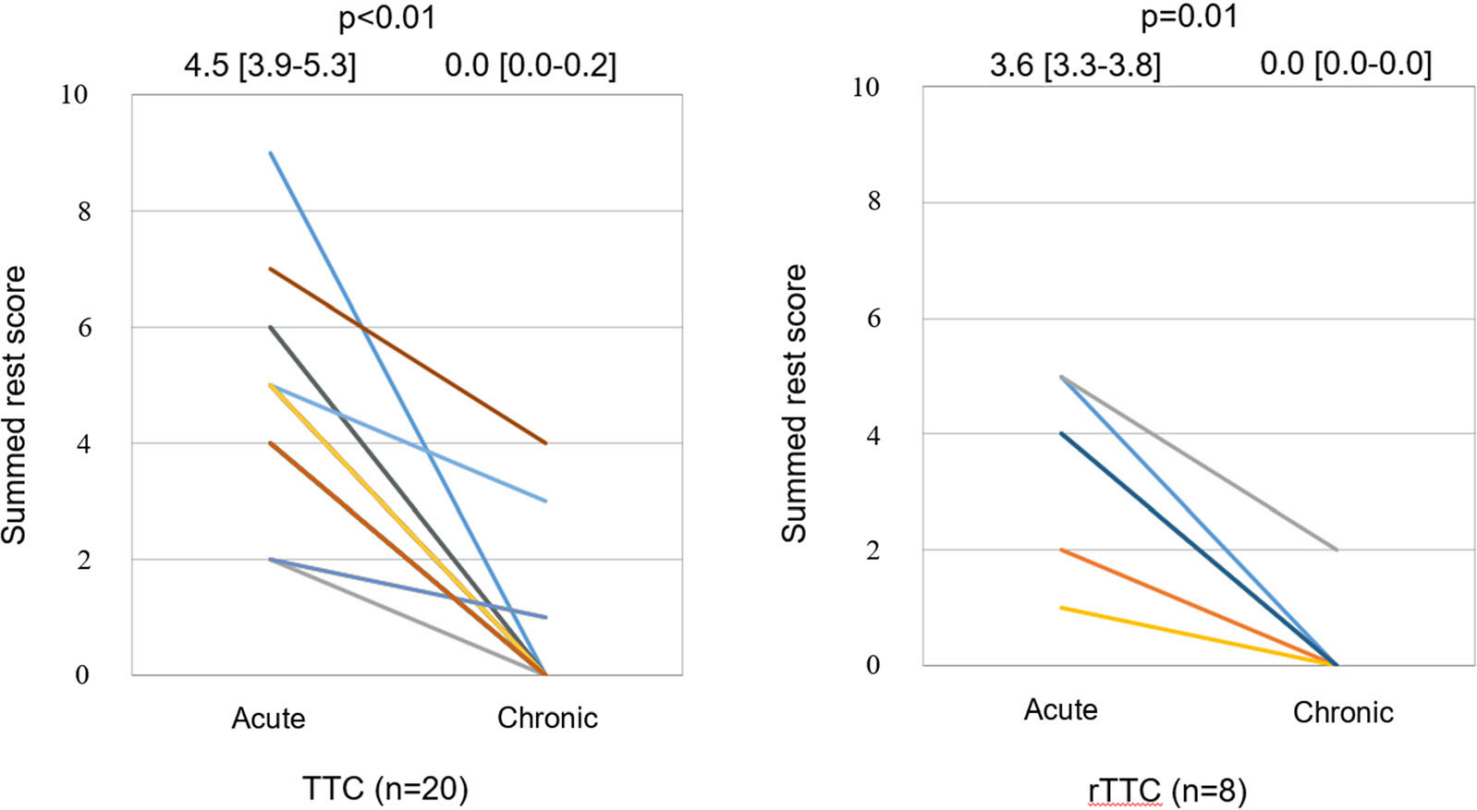
Serial changes of Summed rest score. Summed rest score was also improved from acute to chronic phase in TTC and rTTC group. *TTC*, takotsubo cardiomyopathy; *rTTC*, reverse takotsubo cardiomyopathy

The LVEF in all cases improved as patients progressed from the acute to the chronic phase (TTC: 54 [50-60]% vs 76 [70-83]%, *P* < 0.01; rTTC: 59 [54-61]% vs 65 [63-72]%, *P* < 0.01). The min-%-uptake improved from the acute to the chronic phase (TTC: 54 [48-59]% vs 87 [81-90]%, *P* < 0.01; rTTC: 60 [55-64]% vs 77 [71-79]%, *P* < 0.01) (Figure 6), as did the ES (TTC: 32 [25-40]% vs 0.0 [0.0-6.0]%, *P* < 0.01; rTTC: 16 [11-33]% vs 0.0 [0.0-0.0]%, *P*  = 0.02) (Figure 7) and SRS (TTC: 4.5 [3.9-5.3] vs 0.0 [0.0-0.2], *P* < 0.01; rTTC: 3.6 [3.3-3.8] vs 0.0 [0.0-0.0], *P*  = 0.01) (Figure 8).

## Discussion

The major findings in this study are as follows. (1) The LV function improved one month following hospitalization in both groups. (2) Myocardial perfusion scintigraphy revealed that min-%-uptake, ES, and SRS were mild to moderately reduced in the acute phase in both groups. (3) Min-%-uptake, ES, and SRS were normalized with progression from acute to chronic phase in both TTC and rTTC patients.

Although rTTC is similar to TTC in its clinical course, it is thought to be a variant of TTC. In this study, the distribution of age and gender and the frequency of preceding stressful events in rTTC did not differ from those of TTC, demonstrating similarity between TTC and rTTC. The prevalence of such variants has previously been considered rare, but the reported number of TTC variants has increased in recent years.

Eitel *et al.* reported that cardiovascular magnetic resonance imaging revealed that TTC showed four distinct patterns of regional ventricular ballooning: apical (82%), biventricular (34%), midventricular (17%), and basal (1%).^13^ Templin et al. also reported that apical TTC was identified in 81.7% of subjects, whereas the midventricular form was found in 14.6% and basal and focal forms were diagnosed in 2.2% and 1.5% of subjects, respectively.^11^ Thus, variants of TTC have not been as rare in recent years, but the reason these variants occur is not clear. Although the precise pathophysiological mechanisms of TTC are not understood, it is well known that sympathetic stimulation is likely to be involved. In addition, emotional or physical stress and excessive catecholamine, such as in pheochromocytoma^14^ and subarachnoid hemorrhage,^15^ have been associated with TTC development in many cases.

Nuclear cardiology imaging has provided much information regarding the pathophysiology of TTC. Thallium chloride (201Tl) or Tc-99m sestamibi is used as a tracer for evaluating myocardial blood flow, the myocardial cell membrane, and mitochondrial function. Several studies have shown a decrease in tracer uptake during the acute phase of TTC, and its abnormality gradually improves in the subacute and chronic phases using Tc-99m sestamibi myocardial SPECT.^16^,^17^ This serial change might suggest that coronary microcirculation plays a pathological role as a trigger for myocardial ischemia.^18^ In contrast, it has also been argued that a reduction in perfusion tracer counts occurs because of regional myocardial wall thinning at the apex due to the partial volume effect.^19^ Nuclear cardiology imaging has revealed abnormalities of myocardial metabolism and sympathetic nervous activity in TTC. Myocardial fatty acid metabolism is more severely impaired than myocardial perfusion in TTC using tallium-201 (201Tl) and iodine-123-beta-methyl-p-iodophenyl pentadecanoic acid (123I-BMIPP) SPECT,^20^ and the extent of damage to the myocardial glucose metabolism is more severe than that of myocardial perfusion abnormality.^21^ The discrepancy between myocardial perfusion and fatty acid metabolism appears to improve gradually during follow-up, although the metabolic impairment persists beyond the resolution of the systolic dysfunction.

A positron emission tomography using ^18^F-flourodeoxyglucose (FDG) has shown that glucose metabolism is also severely impaired while myocardial perfusion is almost normal.^21^ This “inverse perfusion-metabolism mismatch” is consistent with post-ischemic myocardial stunning caused by multivessel coronary disease or diffuse coronary microvascular abnormalities.^18^^123^I metaiodobenzyl guanidine (MIBG) scintigraphy can assess myocardial sympathetic nerve terminal activity. Similar defects of MIBG and FDG uptake have been found in the acute and subacute phases, despite slightly reduced perfusion in TTC.^22^ Sympathetic dysfunction is often more extensive than in the region of perfusion and metabolic abnormality in TTC. These observations suggest that the primary cause of TTC may be related to a disturbance in the cardiac sympathetic innervation.^23^ However, the abnormality of MIBG findings may be the result rather than the cause of TTC because the same findings are seen in acute coronary syndrome. A follow-up observation study using ^201^Tl, ^123^IBMIPP, ^123^I-MIBG SPECT showed that the recovery of the decreased uptake mostly occurred in the order of 201Tl, 123I-BMIPP, and 123I-MIBG.^23^-^25^ Moreover, Suzuki *et al.* have measured regional cerebral blood flow, a well-established index of brain activity using ^99m^Tc ethyl cysteinate dimmer SPECT, and demonstrated a significant cerebral blood flow increase in the hippocampus, brainstem, and basal ganglia paralleled by a decrease in the prefrontal cortex in the acute phase of TTC.^26^ Although these changes gradually subsided, they were still present in the chronic phase of TTC even after the cardiac wall motion abnormalities had disappeared. These cerebral blood flow abnormalities may affirm the hypotheses for the pathophysiology of abnormal sympathetic arousal of TTC. Thus, there is much information about the pathophysiology of TTC using nuclear imaging. Therefore, it has been considered that the pathophysiology of TTC is not a single etiology but a combination of several mechanisms in recent years.^27^-^29^

However, there are only a few reports involving nuclear cardiology imaging for the variants of TTC^30^,^31^ where the pathophysiology remains unclear. In this study, in the rTTC group, the decrease of the Tc-99m sestamibi uptake was observed in the area corresponding to the wall movement abnormalities in the acute phase as in the TTC group. In addition, it was clarified that the decrease of Tc-99m sestamibi uptake improved with the normalization of wall motion abnormalities a month after onset of the condition in both groups. These results suggest that the same mechanism may be involved in both TTC and rTTC. Resting myocardial perfusion scintigraphy with Tc-99m sestamibi is a lipophilic cation preparation, and ingestion and retention of myocardium depend on the mitochondrial membrane potential. The defect in the resting imaging indicates damaged myocardium. Therefore, it is thought that the reversible damage of coronary microcirculation and the mitochondria may be involved in the pathophysiology of TTC and rTTC. These findings have been regarded as consistent with coronary microcirculatory disorder and the mitochondrial dysfunction hypothesis of TTC.

Moreover, these findings are similar to those observed in acute myocardial infarction, where reperfusion therapy was performed, and myocardial salvage was successful. In patients with acute anterior infarction, a threshold of 50% of maximal Tc-99m sestamibi uptake had the best predictive value (positive predictive value of 90% and a negative predictive value of 91%) for improvement of LV function.^32^ In this study, the min-%-uptake in the acute phase of TTC and rTTC remained within the predicted range for viability in acute coronary syndrome in most cases. These findings may suggest that damaged myocardia have temporary myocardial ischemia in patients with TTC and rTTC. Multivessel epicardial coronary vasospasms have been suggested as a plausible causative factor of TTC for a long time.^1^,^6^ In our study, the ACh provocation test provoked epicardial coronary spasm in 5 of the 12 (42%) patients. Tawarahara et al. reported a case of TTC in which intracoronary ACh injection induced coronary spasm and the uptake of thallium-201 in the apex was decreased in the acute phase but recovered in the chronic phase.^33^ However, in recent years, TTC involvement and epicardial coronary vasospasm have been denied because provocative vasospasm was confirmed only in a limited number of cases in the ACh provocation test.^25^ Conversely, provoked coronary spasm cases in patients with TTC have been reported and their variants from the early era of discovering this syndrome to the present.^1^,^6^,^33^-^40^ This discrepancy in the relationship between TTC and spasm may be due to the sensitivity of the ACh provocation test. Sueda et al. reported that intracoronary injection of ACh is less sensitive for diagnosis in young patients with rest angina and recommend performing sequential spasm provocation tests of ACh and ergonovine.^41^ Sato et al. reported a case of recurrent takotsubo-like cardiomyopathy associated with pheochromocytoma exhibiting different patterns of LV wall motion abnormality and different results of ACh provocation tests performed several times.^39^ As another possibility, it is hypothesized that epicardial and microvascular spasm could play a pathogenetic role in TTC. Abnormal coronary microvascular responses have been reported with various modalities in TTC. Loffi reported that Thrombolysis in Myocardial Infarction frame count and quantitative blush evaluator was impaired in left anterior descending artery territory in TTC patients.^42^ Some cases also reported that the index of microcirculatory resistance with pressure wire increased in the acute phase and improved in the chronic phase in TTC patients.^43^-^45^ Mildly reduced uptake of Tc-99m sestamibi in TTC and rTTC patients similar to those with stunned myocardium might be due to the coronary microcirculatory disorder and the mitochondrial dysfunction resulting from epicardial or microvascular coronary vasospasm.

### Study Limitations

This study has several limitations. Firstly, it was an observational, retrospective analysis, and a single-center study with a relatively small number of patients. Secondly, this study evaluated only myocardial blood flow using Tc-99m sestamibi but did not evaluate the abnormalities of myocardial metabolism and sympathetic nervous activity associated with TTC and rTTC. Thirdly, this study evaluated only TTC (apical type) and rTTC (basal type), with no other variants (midventricular and focal types). Fourthly, in this study, we evaluated patients who were able to undergo scintigraphy in the acute and chronic phases. Patients who could not perform scintigraphy in the acute phase due to poor general conditions were excluded. LVEF was relatively maintained in both the TTC and rTTC groups, which may have affected the results. Fifthly, there were relatively more asymptomatic patients in our study compared to past cohort studies. Finally, there might be a selection bias because we did not include all TTC patients but only those who had undergone scintigraphy in the acute and chronic phases in this study. Future studies with larger patient cohorts are required to overcome these limitations and to validate our findings.

## New Knowledge Gained

This study was nuclear imaging that investigated TTC and rTTC pathophysiology using Tc-99m sestamibi. Our findings revealed that uptake of Tc-99m sestamibi was similarly reduced in the acute phase but improved in the chronic phase in both TTC and rTTC groups.

Our findings suggest that reversible disorders, such as microcirculation, myocardial cell membrane, and mitochondria may be involved in the pathophysiology of TTC and rTTC.

## Conclusion

The findings on myocardial perfusion imaging of TTC and rTTC are similar to those of stunned myocardium found in acute coronary syndrome. Even in the evaluation of uptake of Tc-99m sestamibi, the degree of reduction in the acute phase remains within the range in which the viability can be predicted in acute coronary syndrome. Our findings suggest that reversible disorders such as microcirculation, myocardial cell membrane, and mitochondria may be involved in TTC and rTTC pathophysiology.

## Supplementary Information

Below is the link to the electronic supplementary material.

Supplementary file1 (PPTX 14273 kb)

## Abbreviations

TTC: Takotsubo cardiomyopathy
rTTC: Reverse takotsubo cardiomyopathy
ES: Extent score
LV: Left ventricular
LVEF: Left ventricular ejection fraction
PER: Peak ejection rate
PFR: Peak filling rate
SPECT: Single photon emission computed tomography
SRS: Summed rest score
