# Supplementary material for: Serial changes of myocardial perfusion imaging in takotsubo and reverse takotsubo cardiomyopathy
Source: J Nucl Cardiol. 2021 Aug 24;29(5):2599–611. doi: 10.1007/s12350-021-02755-y (PMC9553766; doi:10.1007/s12350-021-02755-y)
Supplement: Supplementary file 1 — Supplementary file1 (PPTX 14273 kb) [file 12350_2021_2755_MOESM1_ESM.pptx]

## Slide 1
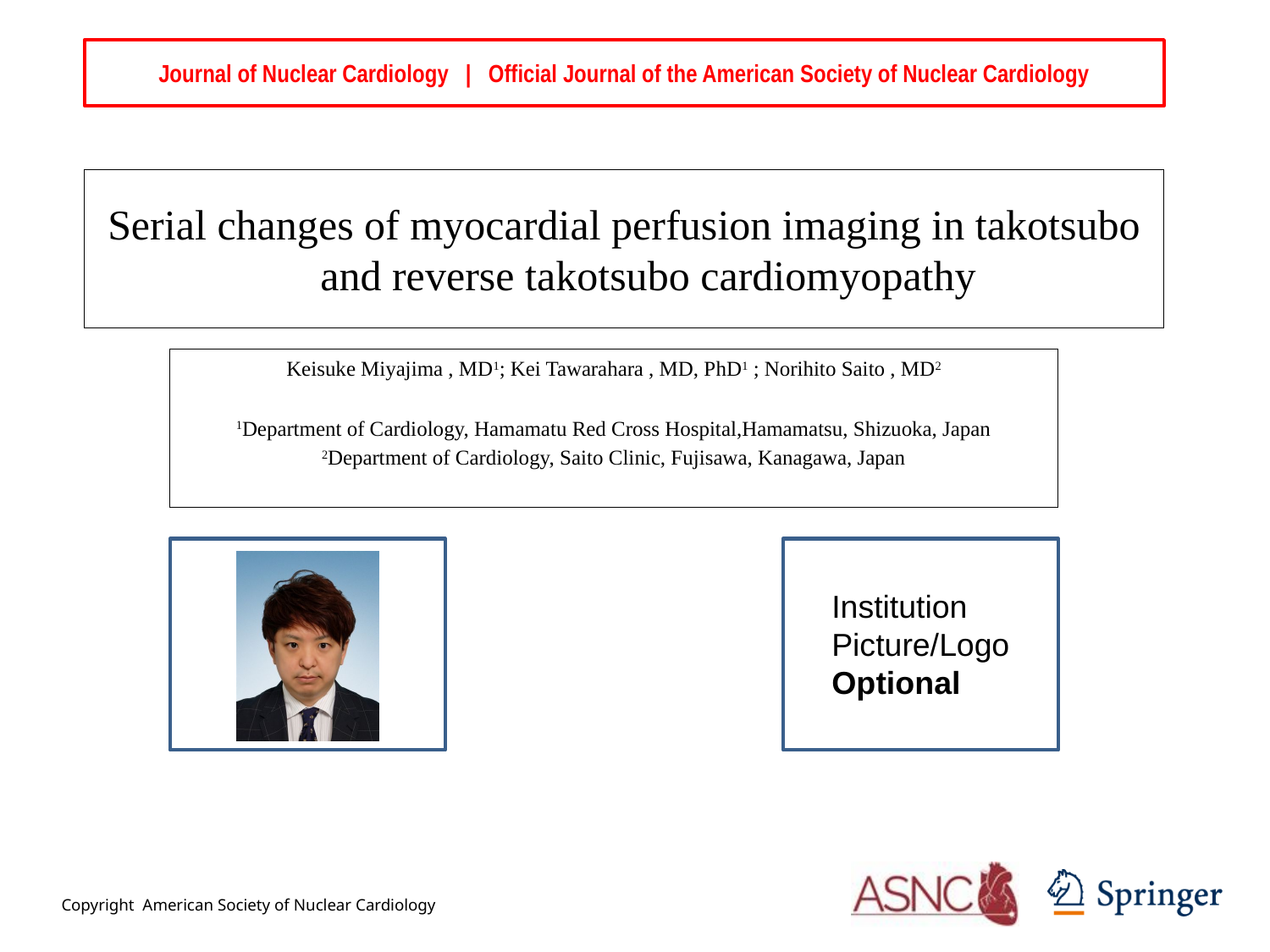

Journal of Nuclear Cardiology | Official Journal of the American Society of Nuclear Cardiology
# Serial changes of myocardial perfusion imaging in takotsubo and reverse takotsubo cardiomyopathy
Keisuke Miyajima , MD1; Kei Tawarahara , MD, PhD1 ; Norihito Saito , MD2
1Department of Cardiology, Hamamatu Red Cross Hospital,Hamamatsu, Shizuoka, Japan
2Department of Cardiology, Saito Clinic, Fujisawa, Kanagawa, Japan
Institution
Picture/Logo
Optional
Copyright American Society of Nuclear Cardiology

## Slide 2
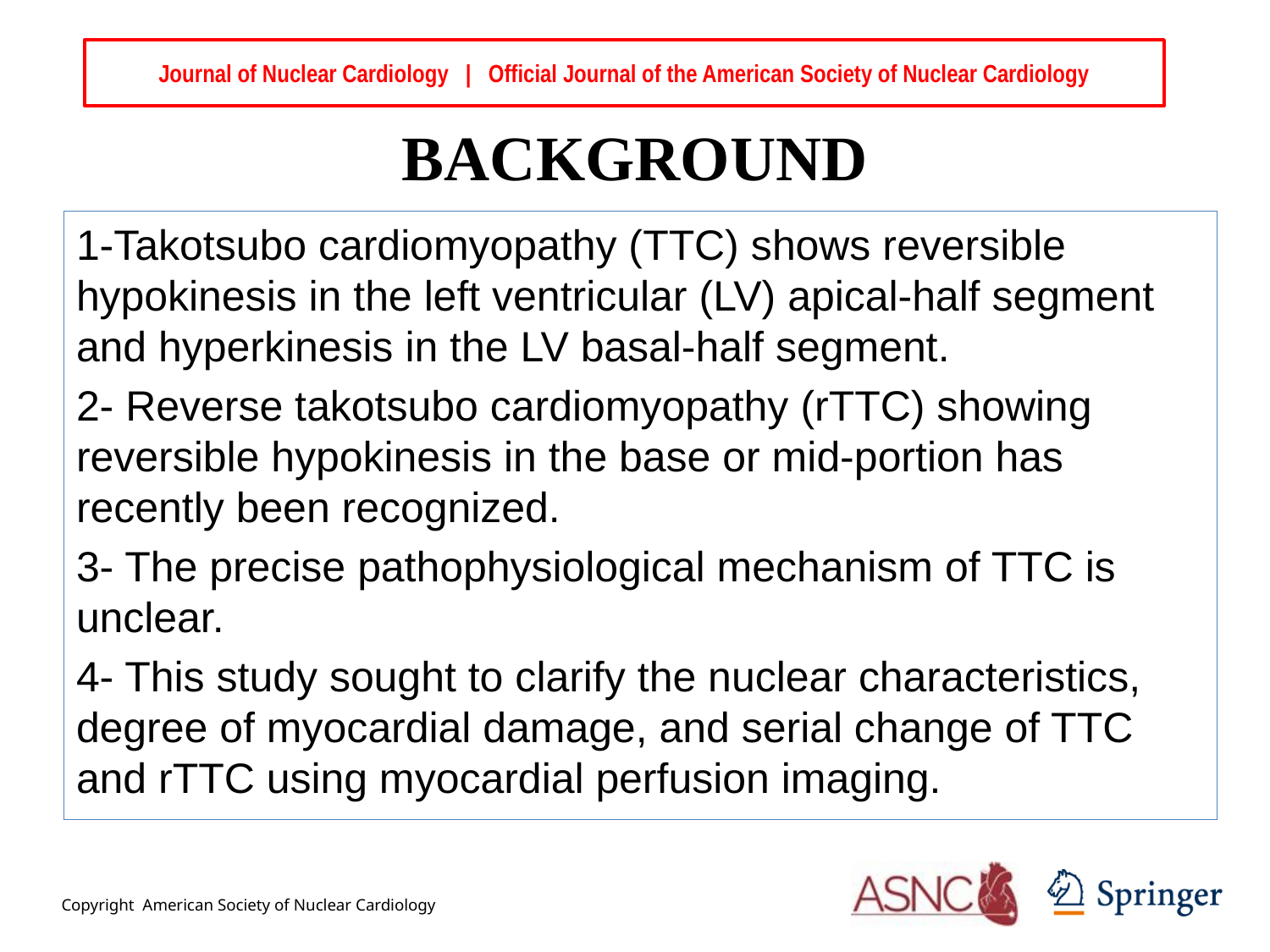

Journal of Nuclear Cardiology | Official Journal of the American Society of Nuclear Cardiology
# BACKGROUND
1-Takotsubo cardiomyopathy (TTC) shows reversible hypokinesis in the left ventricular (LV) apical-half segment and hyperkinesis in the LV basal-half segment.
2- Reverse takotsubo cardiomyopathy (rTTC) showing reversible hypokinesis in the base or mid-portion has recently been recognized.
3- The precise pathophysiological mechanism of TTC is unclear.
4- This study sought to clarify the nuclear characteristics, degree of myocardial damage, and serial change of TTC and rTTC using myocardial perfusion imaging.
Copyright American Society of Nuclear Cardiology

## Slide 3
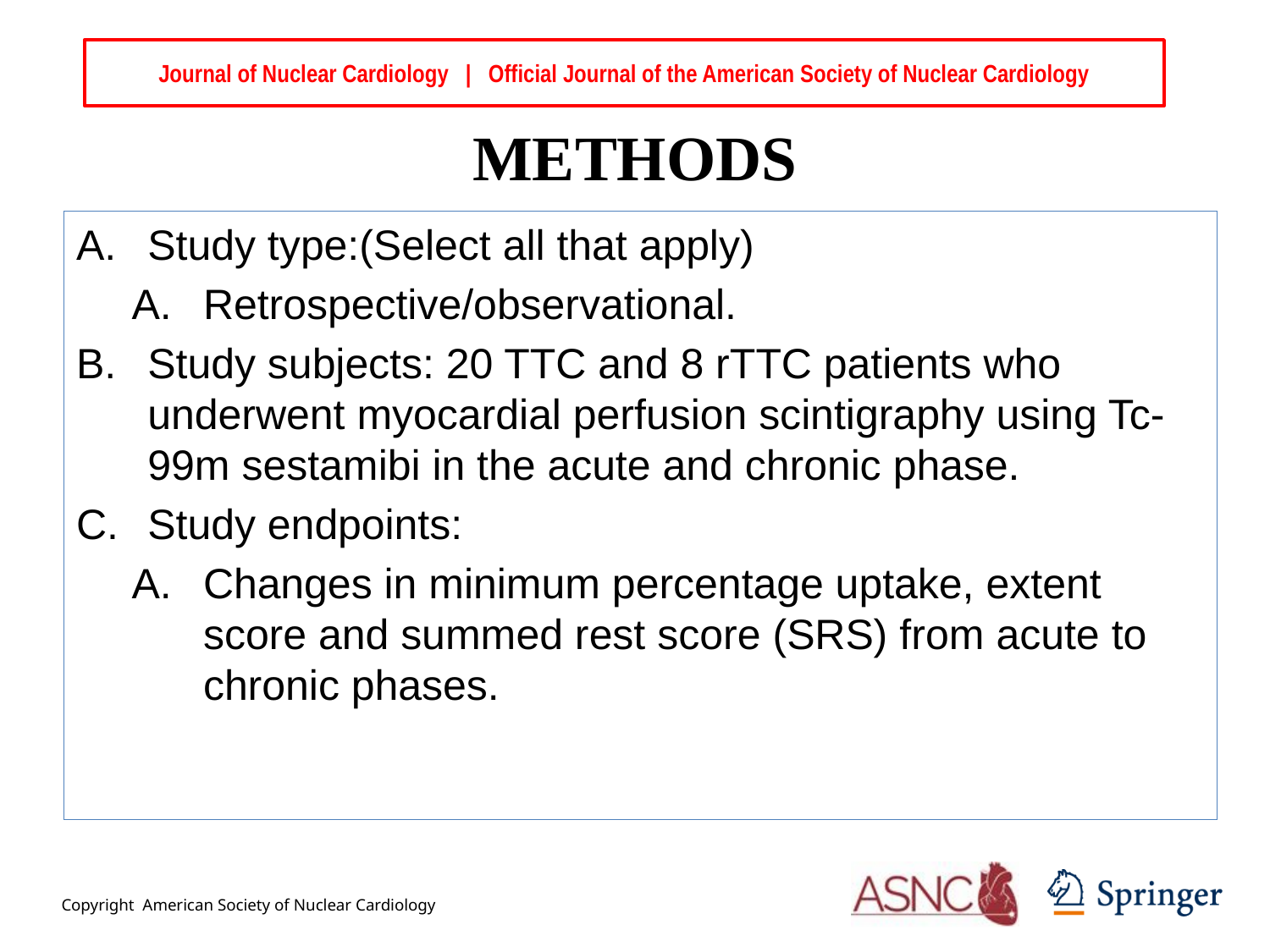

Journal of Nuclear Cardiology | Official Journal of the American Society of Nuclear Cardiology
# METHODS
Study type:(Select all that apply)
Retrospective/observational.
Study subjects: 20 TTC and 8 rTTC patients who underwent myocardial perfusion scintigraphy using Tc-99m sestamibi in the acute and chronic phase.
Study endpoints:
Changes in minimum percentage uptake, extent score and summed rest score (SRS) from acute to chronic phases.
Copyright American Society of Nuclear Cardiology

## Slide 4
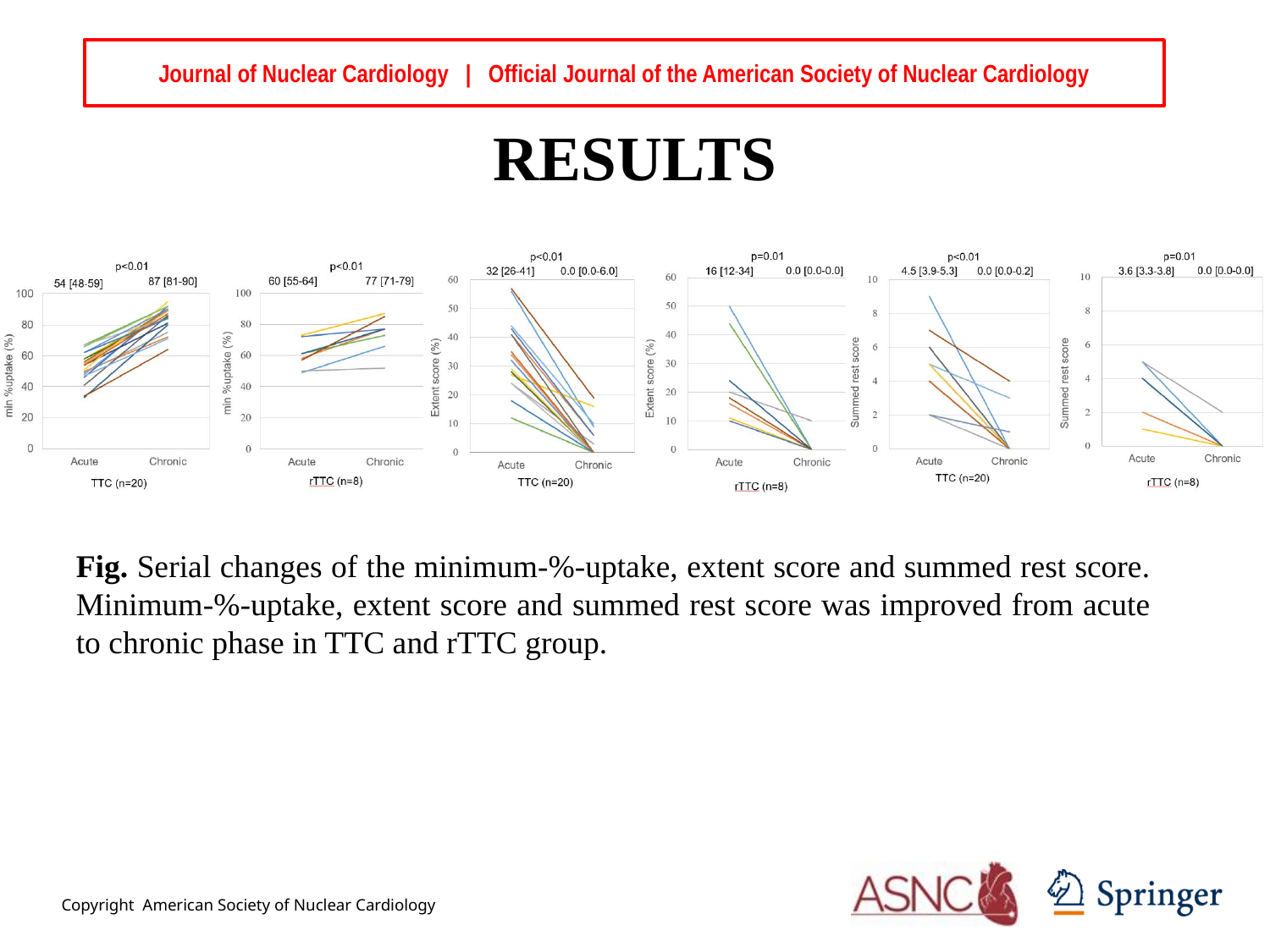

Journal of Nuclear Cardiology | Official Journal of the American Society of Nuclear Cardiology
# RESULTS
Fig. Serial changes of the minimum-%-uptake, extent score and summed rest score. Minimum-%-uptake, extent score and summed rest score was improved from acute to chronic phase in TTC and rTTC group.
Copyright American Society of Nuclear Cardiology

## Slide 5
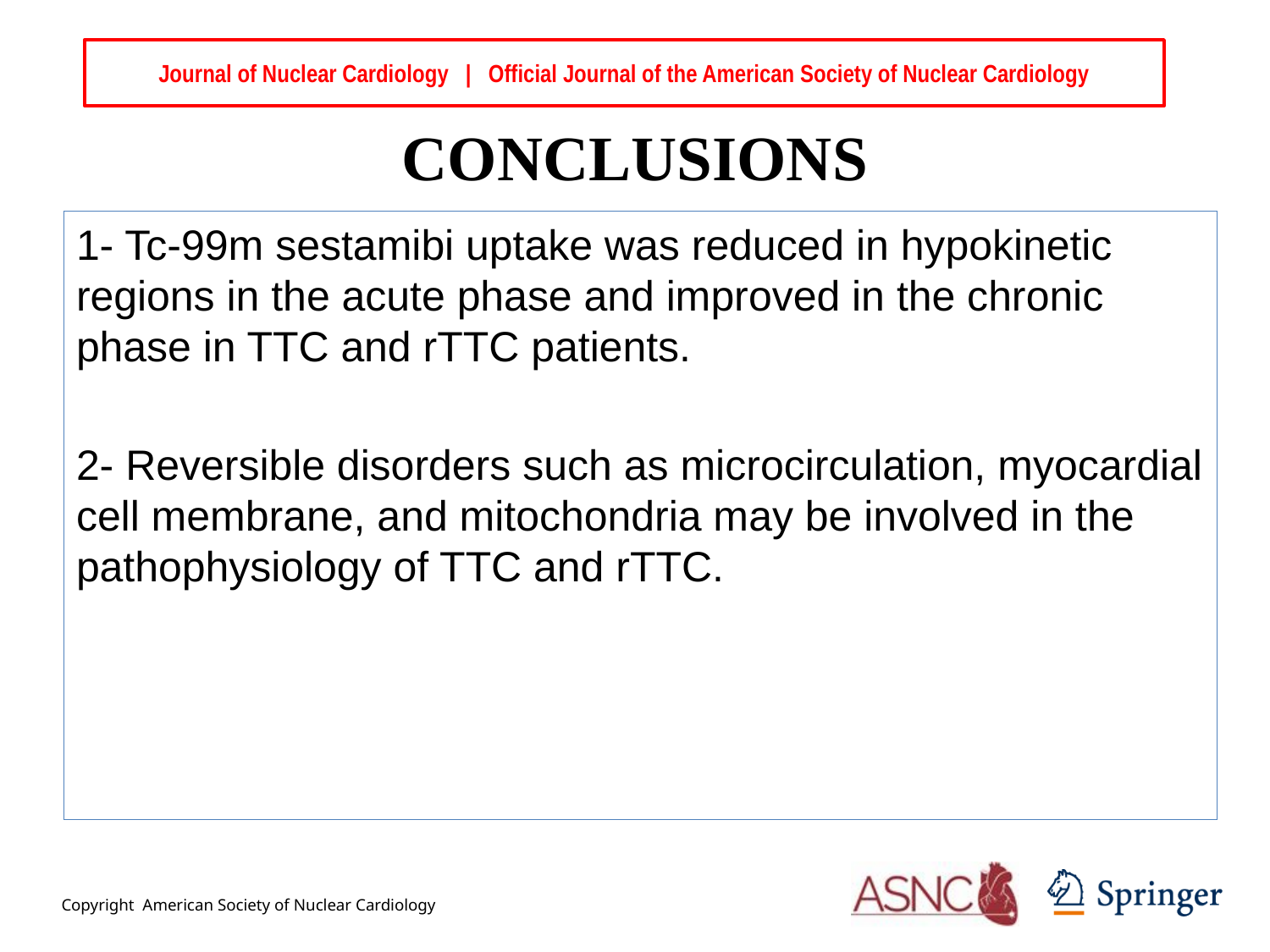

Journal of Nuclear Cardiology | Official Journal of the American Society of Nuclear Cardiology
# CONCLUSIONS
1- Tc-99m sestamibi uptake was reduced in hypokinetic regions in the acute phase and improved in the chronic phase in TTC and rTTC patients.
2- Reversible disorders such as microcirculation, myocardial cell membrane, and mitochondria may be involved in the pathophysiology of TTC and rTTC.
Copyright American Society of Nuclear Cardiology
